# The elevation of different myocardial biomarkers on admission is associated with disease features and different outcomes in aneurysmal subarachnoid hemorrhage

**DOI:** 10.1038/s41598-022-20650-5

**Published:** 2022-10-05

**Authors:** Yuqi Chen, Chengzhi Cai, Jiang Fei, Song Luo, Chao You

**Affiliations:** 1 Department of Neurosurgery, West China Hospital, Sichuan University, 37 Guoxue Alley, Chengdu, 610041 Sichuan People’s Republic of China; 2 West China School of Medicine, Sichuan University, 37 Guoxue Alley, Chengdu, 610041 Sichuan People’s Republic of China; 3 The First Affiliated Hospital, Zhejiang University School of Medicine, 79 Qingchun Road, Hangzhou, 310003 Zhejiang People’s Republic of China

**Keywords:** Biomarkers, Diseases, Medical research, Neurology

## Abstract

Test of different myocardial biomarkers is commonly arranged in patients with aneurysmal subarachnoid hemorrhage (aSAH). We sought to figure out whether different myocardial biomarkers' elevation is related to characteristics of ruptured aneurysms and patients' clinical outcomes. Patients with aSAH admitted in the Neurosurgery Department of West China Hospital from September 2019 to March 2020 were screened. Those who have one clear responsible aneurysm and met inclusion criteria were included. Clinical characteristics, site and size of the aneurysm, modified Fisher scale, troponin T (TPN-T), creatine kinase MB (CK-MB), and myoglobin (Myo) levels at admission, clinical outcomes (3-month mRS) were collected and compared. The study included 124 patients. After multivariate logistic regression, Hunt & Hess grade (per unit grade, OR 1.68, 95% CI 1.14–2.49), the size of ruptured aneurysm (equal to or more than 7 mm, OR 3.07, 95% CI 1.32–7.10) was highly predictive of myocardial biomarker elevation. All three biomarkers (TPN-T, CK-MB, Myo) were associated with unfavorable prognoses. Higher mortality (37.2% vs. 18.6%, *P* = 0.036) and a lower rate of good outcomes (41.9% vs. 71.2%, *P* = 0.003) were observed in patients with any positive myocardial biomarkers at admission. The clinical outcomes of patients with positive troponin T and negative creatine kinase MB were especially unfavorable. Our study demonstrates that the degree of neurological injury and size of ruptured aneurysm are strong predictors of myocardial biomarkers elevation, the site of ruptured aneurysm may not be associated with heart injury after SAH. The outcomes of patients with different combinations of abnormal biomarker levels may have significant differences and deserve further study.

## Introduction

Aneurysmal subarachnoid hemorrhage (aSAH) is a common type of hemorrhagic stroke with notable mortality^1^. As the mean age of aneurysmal rupture is in the range of 50 to 55 years^2^, physical and psychological sequelae following aSAH also seriously affect patients' quality of life^3^. Myocardial biomarkers, including troponin T (TPN-T), creatine kinase MB (CK-MB), and myoglobin (Myo), have been commonly used in the early diagnosis of myocardial infarction (MI)^4^. Troponin T is the most sensitive and specific index for MI; however, the time window limits its application. The elevation of TPN-T could be detected several hours later after MI. For this reason, the comprehensive detection of 3 kinds of biomarkers is helpful to diagnosing MI as soon as possible^5^. Myocardial biomarkers elevation in aneurysmal subarachnoid hemorrhage (aSAH) has been observed for a long time, but the mechanisms are still poorly understood^6,7^. The likely explanations include "demand ischemia" (which is also known as "type 2 MI") and "imbalance of the autonomic nervous system"^8,9^. Most former studies focused on cardiac troponin and found that troponin's elevation is associated with an increased risk of delayed cerebral infarction, poor clinical outcomes, and death^10,11^. Other research showed that clinical characteristics such as Hunt-Hess score, blood pressure, sex, body surface area, and heart rate were independent predictors of troponin elevation^12^. However, limited studies focused on aneurysm's characteristics (site, size, etc.) or tried to use cardiac biomarkers in aSAH comprehensively. In this study, we sought to determine whether myocardial biomarkers' elevation is related to characteristics of ruptured aneurysms. We also attempted to figure out whether myocardial biomarker elevation (not just TPN-T elevation) on admission is associated with unfavorable clinical outcomes.

## Materials and methods

From September 2019 to March 2020, all patients with SAH who visited the Neurosurgery Department of West China Hospital were retrospectively screened. Our study's inclusion criteria include: 1. age > 18 years old; 2. confirmed diagnosis of SAH by non-contrast head CT, and established diagnosis of aneurysm by CTA or DSA; 3. only one ruptured aneurysm with clear description about size and site was found (patients with multiple aneurysms or no evidence of aneurysm were excluded from our study); 4. serum concentration of myocardial biomarkers (Myo, CK-MB and TPN-T) was measured on admission, and the results are available (in our hospital, all aSAH patients were routinely scheduled for tests of these biomarkers within 24 h of admission). Patients with diseases that could lead to long-term elevation of any myocardial biomarkers were excluded. The exclusion of patients depends on the history and result of former myocardial biomarkers’ test. If a specific patient has chronic disease, and the serum levels of biomarkers were detected to increase (before admission for aSAH), then the patient will be excluded. Serum levels of Myo, CK-MB, TPN-T were measured by ELISA using a VIDAS analyzer (BioMerieux, France). In our hospital, Myo equal to or more than 58 ng/ml, CK-MB equal to or more than 2.88 ng/ml, TPN-T more than 14 ng/L were considered abnormal. For eligible patients, their medical records, including age, sex, vascular risk factors (drinking and smoking), underlying diseases (especially history of cardiopulmonary disease), blood pressure on admission, baseline Hunt & Hess grade, imaging studies (CTA or DSA), treatment, do-not-rescue (DNR) order, and clinical outcomes (modified Rankin Scale, mRS) were reviewed and recorded. In this study, both former and current smokers are defined as smokers. Drinkers are defined as those who have consumed alcohol more than the amounts that increase health risks, refer to the estimation from The National Institute on Alcohol Abuse and Alcoholism (NIAAA). In imaging analysis, we collected information about the ruptured aneurysm (site, size) and evaluated the range of bleeding, modified Fisher scale (Zervas et al., 1997) was graded. When we recorded sites of aneurysms, they were classified to anterior and posterior circulation aneurysms. Except for common detailed position, some aneurysms were classified into “other anterior” group (such as anterior choroidal, and ophthalmic aneurysms), some aneurysms were classified into “other posterior” group (such as anterior inferior cerebellar artery aneurysm, and posterior inferior cerebellar artery aneurysm). The DNR order was defined as any refusal of necessary treatment (for example, decline transfer to intensive care unit, request immediate discharge, decline resuscitation). All patients were followed up through phone calls, mRS at 3 months was recorded to assess the clinical outcome, good outcome was defined as an mRS of 0–2 at the follow-up.

Our data were analyzed by SPSS statistics 23.0 (IBM SPSS Statistics, Armonk, NY, USA). Independent-samples *t* tests, chi-square tests, Mann–Whitney U tests were performed where appropriate for the comparison of different types of data. We also performed multivariate logistic regression to quantify the relationships between clinical characteristics and myocardial biomarkers' elevation. After we transferred the test results of myocardial biomarkers to numerical values (positive = 1, negative = − 1) and performed binary logistic regression, receiver-operating characteristic (ROC) analysis was performed to evaluate unfavorable outcome predictability of two different ways (only use TPN-T or use all biomarkers). *P* values less than 0.05 were considered statistically significant.

### Ethical approval

This study has been performed in accordance with the Declaration of Helsinki. Prior to conducting the study, approval was obtained from the Ethics Council of West China Hospital, Sichuan University. As a retrospective study, no written consent was required according to the review from the Ethics Council of West China Hospital, Sichuan University.

## Results

**Figure 1:**
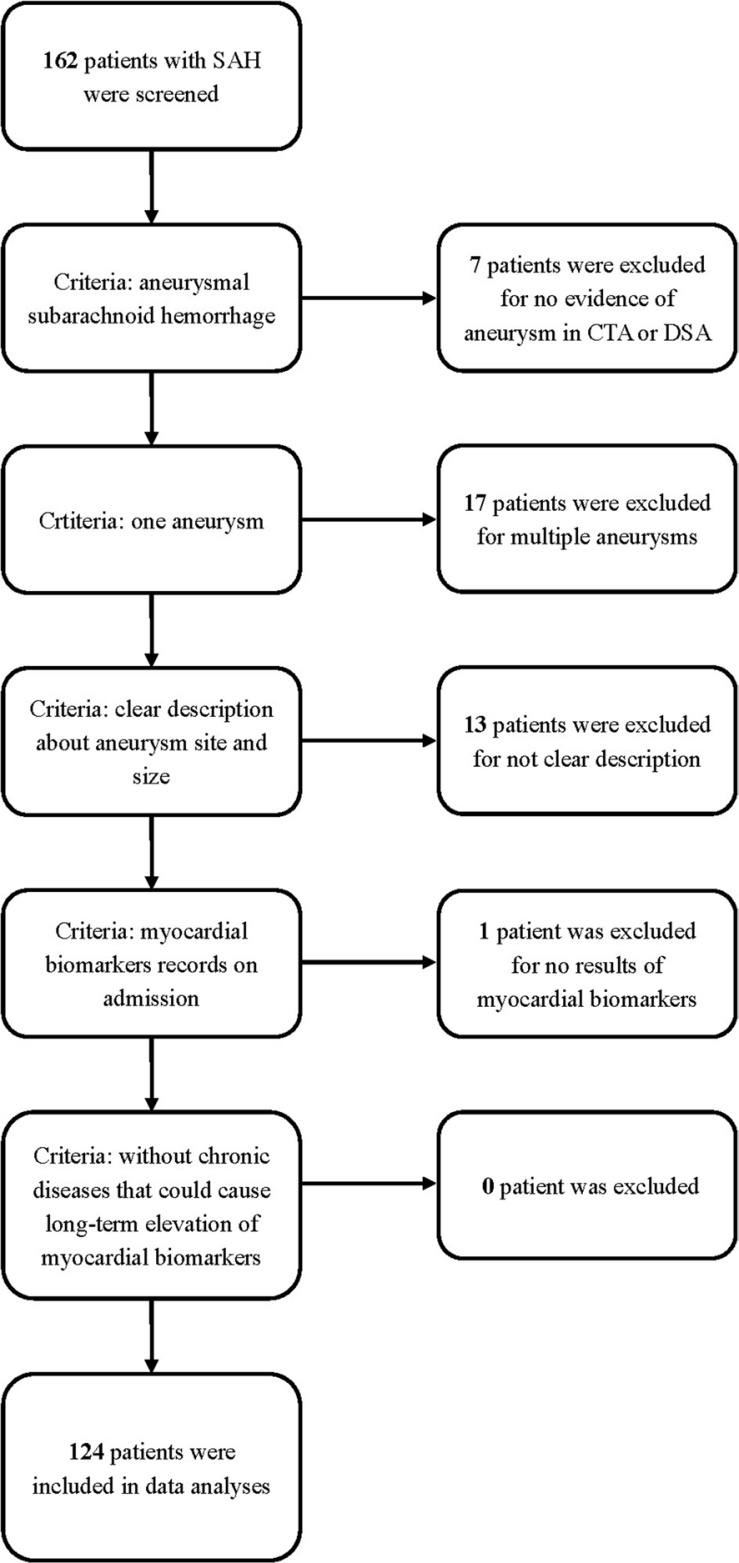
Patients selection flow chart.

Between September 2019 to March 2020, 162 patients with subarachnoid hemorrhage visited the Neurosurgery Department of West China Hospital, 124 patients fulfilled all inclusion criteria and were included in our study, our screening procedures are presented in Fig. 1. The clinical characteristics of patients with or without myocardial biomarker elevation were summarized and compared in Table 1. It has to be stated that “heart disease” in Table 1 includes tachycardia, aortic valve replacement, stable ischemic heart disease, and premature atrial contractions; “pulmonary disease” includes chronic obstructive pulmonary disease (without heart failure) and pneumoconiosis; “hepatic disease” includes hepatitis B and autoimmune hepatitis. There were no significant differences in age, sex, vascular risk factors (smoke, drink, hypertension), or underlying diseases between the two groups. When compared the blood pressure at admission, median diastolic blood pressure (DBP) in patients without myocardial biomarker elevation was higher than that in patients with myocardial biomarker elevation, but no statistical difference was observed (84.0 [73.3–100.8] *vs.* 89.5 [78.3–99.8], P = 0.160). The disease severity (Hunt & Hess grade) for patients with myocardial biomarker elevation was greater than that for patients without abnormality (3 [3–4] *vs.* 3 [2–3], *P* = 0.001). More patients with myocardial biomarker elevation were graded 3–4 in modified Fisher scale (86.5% *vs.* 66.7%, *P* = 0.012), while the rate of intracerebral hemorrhage (ICH) or intraventricular hemorrhage (IVH) is similar between the two groups. The detailed site of ruptured aneurysm of each patient was presented in Table 1. Overall, more anterior circulation aneurysms were reported, no specific sites were found to be strongly related to the abnormal myocardial biomarker level in our research. It's worth to note that the proportion of large size (equal to or more than 7 mm) ruptured aneurysm in patients with abnormal myocardial biomarkers was significantly higher than that in patients without myocardial biomarker elevation (44.2% *vs.* 18.1%, *P* = 0.002). From the perspective of treatment decisions, there were no significant differences between the two groups.

**Table 1:** Clinical Characteristics of Enrolled Patients.

	Patients with one or more myocardial biomarkers elevation (N = 52)	Patients without myocardial biomarker elevation (N = 72)	*P* Value
Mean age, y (SD)	57.7 (11.1)	55.1 (12.0)	0.230
Males, %	19 (36.5)	28 (38.9)	0.790
Smoker, %	7 (13.5)	12 (16.7)	0.625
Drinker, %	4 (7.7)	11 (15.3)	0.201
Hypertension	23	34	0.742
Diabetes mellitus	2	2	1.000
Renal insufficiency	2	0	0.174
Heart disease	1	2	1.000
Pulmonary disease	2	3	1.000
Hepatic disease	0	3	0.264
Median-Systolic blood pressure (SBP) at admission (IQR)	143.0 (123.8–158.3)	148.0 (128.0–160.0)	0.328
Median-Diastolic blood pressure (DBP) at admission (IQR)	84.0 (73.3–100.8)	89.5 (78.3–99.8)	0.160
Median-Hunt & Hess grade (IQR)	3 (3–4)	3 (2–3)	0.001
**Modified Fisher scale**			
0–2	7 (13.5)	24 (33.3)	0.012
3–4	45 (86.5)	48 (66.7)	
With IVH, %	29 (55.8)	32 (44.4)	0.213
With ICH, %	6 (11.5)	10 (13.9)	0.700
**Site of ruptured aneurysm**			
Anterior circulation aneurysm	46	65	0.745
AComA	12	20	
ACA	2	4	
MCA	8	14	
ICA	19	20	
PComA	5	7	
Other anterior	0	0	
Posterior circulation aneurysm	6	7	
Vertebral artery	4	2	
Basilar apex	0	1	
PCA	0	2	
Other posterior	2	2	
**Size of ruptured aneurysm, mm**			
< 7	29 (55.8)	59 (81.9)	0.002
≥ 7	23 (44.2)	13 (18.1)	
Surgical treatment, %	35 (67.3)	58 (80.6)	0.800
Microsurgical clipping	30	50	
Endovascular occlusion	5	8	
DNR order at any stages of treatment, %	18 (34.6)	17 (23.6)	0.179

*ACA* anterior cerebral artery, *AComA* anterior communicating artery, *DNR* do not rescue, *ICA* internal carotid artery, *ICH* intracerebral hemorrhage, *IVH* intraventricular hemorrhage, *MCA* middle cerebral artery, *PCA* posterior cerebral artery, *PComA* posterior communicating artery.

Based on the sample size and results from univariate comparison, five variables showed statistically significant differences in the former analysis (Hunt & Hess grade, size of ruptured aneurysm, modified Fisher scale 3–4) or had clinical significance (IVH, ICH) were selected for multivariate logistic regression (Table 2). After statistical adjustment, Hunt & Hess grade (per unit grade, OR 1.68, 95% CI 1.14–2.49), the size of ruptured aneurysm (equal to or more than 7 mm, OR 3.07, 95% CI 1.32–7.10) was highly predictive of myocardial biomarker elevation. modified Fisher scale 3–4, IVH, ICH were not included in the predictive equation anymore.

**Table 2:** Multivariable logistic regression model results of myocardial biomarker elevation.

Predictors	Univariate		Multivariate	
	OR (95% CI)	*P* Value	OR (95% CI)	*P* Value
**Variables in the Equation**				
Hunt & Hess grade, per unit grade	1.80 (1.24–2.62)	0.002	1.68 (1.14–2.49)	0.008
Size of ruptured aneurysm, ≥ 7 versus < 7 mm	3.60 (1.60–8.11)	0.002	3.07 (1.32–7.10)	0.009
**Variables not in the Equation**				
Modified Fisher scale 3–4	3.21 (1.26–8.19)	0.014		0.068
IVH	1.58 (0.77–3.23)	0.214		0.517
ICH	0.809 (0.27–2.39)	0.700		0.291

*ICH* intracerebral hemorrhage, *IVH* intraventricular hemorrhage.

**Figure 2:**
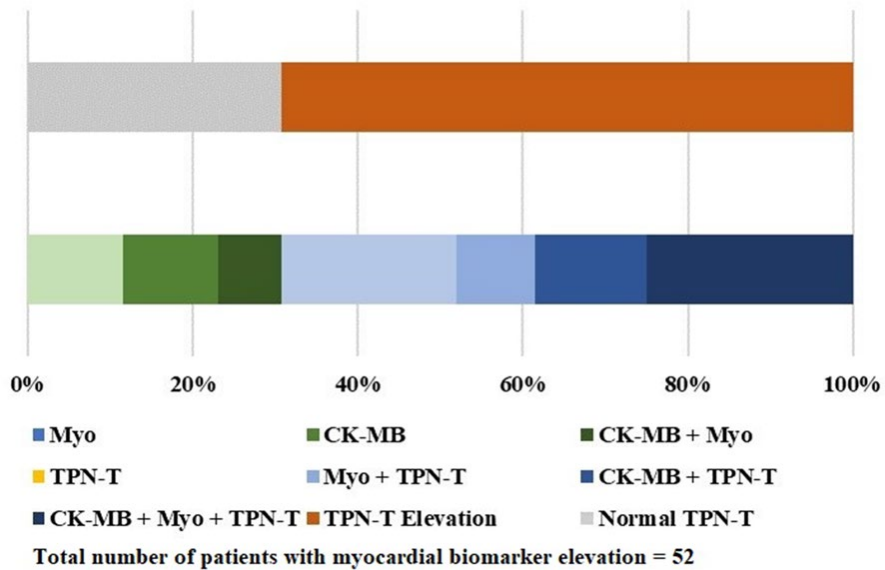
Different combinations of elevated myocardial biomarkers.

For 52 patients with myocardial biomarker elevation, the distribution of different combinations of abnormal myocardial biomarkers was summarized in Fig. 2. Nearly seventy percents of patients with myocardial biomarker elevation have change in TPN-T. About a quarter of these patients have abnormal CK-MB, Myo and TPN-T levels at the same time. No kind of combination took over the absolute predominance.

The clinical outcomes of 102 patients were gathered at three months (Table 3). When compared with all negative patients, all three biomarkers were associated with unfavorable outcomes at three months (TPN-T, 35.5% *vs.* 71.2%, *P* = 0.001; CK-MB, 48.0% *vs.* 71.2%, *P* = 0.043; Myo, 45.8% *vs.* 71.2%, *P* = 0.029), but a statistically significant difference in mortality was only found in positive TPN-T (*P* = 0.018). In patients without myocardial biomarker elevation, the percentage of patients who achieved good outcomes was much higher than that in patients with myocardial biomarker elevation (41.9% *vs.* 71.2%, *P* = 0.003), lower mortality was also observed (37.2% *vs.* 18.6%, *P* = 0.036). Among all different combinations, the clinical outcomes of patients who only have abnormal TPN-T levels and patients who have abnormal Myo and TPN-T levels were worst; 66.7% and 75% of these patients died in three months separately. When using positive TPN-T to predict the unfavorable outcomes, sensitivity was 47.6%, specificity was 81.7%; when using all biomarkers, sensitivity was 59.5%, specificity was 70.0%. According to the results of ROC analysis, the area under curve (AUC) of "only TPN-T" was 0.646 (95% CI 0.535–0.758, *P* = 0.012), which was smaller than that of "all biomarkers" (AUC = 0.652, 95% CI 0.539–0.765, *P* = 0.009).

**Table 3:** Clinical outcomes.

Types				Number	Outcome collected	Good outcome,%		3-month mortality,%
TPN-T	CK-MB	Myo						
Positive	Positive	Positive		13	12	5 (41.7)		3 (25.0)
		Negative		7	6	3 (50.0)		1 (16.7)
	Negative	Positive		5	4	1 (25.0)		3 (75.0)
		Negative		11	9	2 (22.2)		6 (66.7)
Negative	Positive	Positive		4	3	2 (66.7)		1 (33.3)
		Negative		6	4	2 (50.0)		1 (25.0)
	Negative	Positive		6	5	3 (60.0)		1 (20.0)
		Negative		72	59	42 (71.2)		11 (18.6)
Positive	Positive or Negative	Positive or Negative		36	31	11 (35.5)		13 (41.9)
***P* Value (compared with all negative)**						0.001		0.018
Positive or Negative	Positive	Positive or Negative		30	25	12 (48.0)		6 (24.0)
***P* Value (compared with all negative)**						0.043		0.576
Positive or Negative	Positive or Negative	Positive		28	24	11 (45.8)		8 (33.3)
***P* Value (compared with all negative)**						0.029		0.149
Any Positive				52	43	18 (41.9)		16 (37.2)
***P* Value (compared with all negative)**						0.003		0.036
Total				124	102	60 (58.8)		27 (26.5)

## Discussion

In this study, we creatively tried to analyze the relationships between different combinations of abnormal myocardial biomarkers levels and patients' clinical features. According to the guideline, an immediate test of troponin for the evaluation of ischemic and hemorrhagic stroke is recommended^13^. In clinical practice, Myo and CK-MB's tests would be arranged at the same time for better accuracy. Our research would help the better application of myocardial biomarkers in patients with aSAH.

When compared the diastolic blood pressure (DBP) at admission from two different groups of patients, we found the numeric value of patients with myocardial biomarkers elevation is lower than that of patients without such change. Although there was no statistically significant difference, the tendency is compatible with the necessary knowledge that the heart muscle's blood supply is mainly from the coronary artery during the diastolic phase. As type 2 MI could be caused by ischemia, lower DBP adversely affects the heart's blood supply, so it's a reasonable explanation for myocardial biomarkers elevation. Maybe our sample size is too small to show a significant effect. In research of hemorrhagic stroke, we always pay more attention to SBP but not DBP, but understand the role of DBP through reliable theory is also important.

In the present study, Hunt & Hess grades, size of ruptured aneurysms were independent predictors of myocardial biomarker elevation. Many researchers have reported the relationship between higher Hunt & Hess grades and higher troponin levels. Through this study, our results extend our understanding of Hunt & Hess grades that, when analyzed comprehensively, the association remained after we change troponin to any myocardial biomarkers. Interestingly, there was a difference in the size of ruptured aneurysms between the two groups. According to UCAS research, 7 mm is the cutoff value for predicting aneurysm rupture^14^, so we also used 7 mm as the cutoff value in our study. Our research is the first study that reported the association between the size of ruptured aneurysms and myocardial biomarker elevation to the best of our knowledge.

Before performing this study, we assumed that there would be a difference in the distribution of the sites of aneurysms. This assumption comes from functional dissection of innervation of the heart^15^ and the theory mentioned before that sympathetic nervous system activation causes the change of myocardial biomarkers^16^. Since cortex, subcortical forebrain, brain stem all play roles in the central reflex and sympathetic control of the heart, it's reasonable to find a difference when an aneurysm's rupture affects different parts of the brain. However, no statistically significant difference was observed, whether in the site of the aneurysm or the rate of IVH, ICH. A higher rate of modified Fisher scale 3–4 was observed in patients with myocardial biomarker elevation; this could be explained by bleeding affects the cortex, but modified Fisher scale 3–4 is not an independent predictor after statistical adjustment. We tried to provide more support from clinical data to sympathetic nervous system activation theory, but it failed. More research is needed to answer why myocardial biomarkers could elevate in patients with hemorrhagic stroke.

For patients with myocardial biomarker elevation, the higher 3-month mortality and a lower rate of good outcomes are consistent with the tendency of former studies that only focused on troponin^10,11,17^. In our research, the outcomes of patients with different combinations of abnormal myocardial biomarker levels were analyzed separately. It is worth noting that the unfavorable outcomes of patients with myocardial biomarker elevation mainly come from patients with TPN-T change, specifically those patients who only have TPN-T change (22.2% good outcome) or have all biomarkers' change (25.0% good outcome). Why normal CK-MB level at admission for patients with TPN-T change seems like a "protective factor" is hard to explain. One study from GRACE research has described the relationship between CK-MB levels and clinical outcomes in patients with acute coronary syndrome (ACS); they found patients with positive troponin and positive CK-MB have the worst outcome, while patients with negative troponin and negative CK-MB achieved the lowest in-hospital mortality^18^. These results in ACS (Type 1 MI) are in contrast with our results in SAH (maybe can be explained by type 2 MI).

There are some limitations in our study. First, this is retrospective research from a single-center, so the perspectives and quality of medical information are naturally limited. Second, because we only included patients with one clear responsible aneurysm to avoid confounding factors, the total sample size is a little bit insufficient, although our center has a big patient group every year. Third, we have not collected continuous data of myocardial biomarkers; thus, the peak level of these indicators is unavailable in our study. Of course, the time of disease onset will affect the detected level of biomarkers; compared with the level at admission, the peak level is more credible. But continuous tests has not been recommended in patients with normal admission level, and it may add unnecessary burden to our patients.

## Conclusion

In conclusion, our study demonstrates that the degree of neurological injury and size of ruptured aneurysm are strong predictors of myocardial biomarkers elevation, the site of ruptured aneurysm may not be associated with heart injury after SAH. The outcomes of patients with different combinations of abnormal biomarker levels may have significant differences and deserve further study.

## Data Availability

The datasets used and/or analysed during the current study available from the corresponding author on reasonable request.
